# Comparing the Efficacy of MALDI-TOF MS and Sequencing-Based Identification Techniques (Sanger and NGS) to Monitor the Microbial Community of Irrigation Water

**DOI:** 10.3390/microorganisms11020287

**Published:** 2023-01-21

**Authors:** Botond Bendegúz Surányi, Benjamin Zwirzitz, Csilla Mohácsi-Farkas, Tekla Engelhardt, Konrad J. Domig

**Affiliations:** 1 Department of Food Microbiology, Hygiene and Safety, Hungarian University of Agriculture and Life Sciences, Somlói Street 14–16, 1118 Budapest, Hungary; 2 Institute of Food Science, Department of Food Science and Technology, University of Natural Resources and Life Sciences, Vienna, Muthgasse 18, 1190 Vienna, Austria; 3 Digital Food Institute, University of Veterinary Medicine, István Street 2, 1078 Budapest, Hungary

**Keywords:** MALDI-TOF MS, Sanger sequencing, 16S rRNA gene amplicon sequencing, irrigation water, microbial monitoring

## Abstract

In order to intensify and guarantee the agricultural productivity and thereby to be able to feed the world’s rapidly growing population, irrigation has become very important. In parallel, the limited water resources lead to an increase in usage of poorly characterized sources of water, which is directly linked to a higher prevalence of foodborne diseases. Therefore, analyzing the microorganisms or even the complete microbiome of irrigation water used for food production can prevent the growing numbers of such cases. In this study, we compared the efficacy of MALDI-TOF Mass spectrometry (MALDI TOF MS) identification to 16S rRNA gene Sanger sequencing of waterborne microorganisms. Furthermore, we analyzed the whole microbial community of irrigation water using high-throughput 16S rRNA gene amplicon sequencing. The identification results of MALDI-TOF MS and 16S rRNA gene Sanger sequencing were almost identical at species level (66.7%; 64.3%). Based on the applied cultivation techniques, *Acinetobacter* spp., *Enterobacter* spp., *Pseudomonas* spp., and *Brevundimonas* spp. were the most abundant cultivable genera. In addition, the uncultivable part of the microbiome was dominated by Proteobacteria followed by Actinobacteria, Bacteroidota, Patescibacteria, and Verrucomicrobiota. Our findings indicate that MALDI-TOF MS offers a fast, reliable identification method and can act as an alternative to 16S rRNA gene Sanger sequencing of isolates. Moreover, the results suggest that MALDI-TOF MS paired with 16S rRNA gene amplicon sequencing have the potential to support the routine monitoring of the microbiological quality of irrigation water.

## 1. Introduction

Recycled and microbiologically non-characterized waters are increasingly used as irrigation water in agriculture in order to cope with water limitation due to climate change and to support rapid population growth. Crops can be contaminated with potentially harmful microorganisms at any of the several steps in the food production chain, during primary production, at processing stage, and during preparation as in each step water plays a crucial role. At farm level, one of the major sources of foodborne pathogens is insufficient-quality irrigation water, which can be contaminated by sewage overflows or polluted storm and agricultural runoffs [1,2]. Since many bacterial pathogens (*Listeria monocytogenes*, verotoxigenic *Escherichia coli*, *Salmonella* spp., *Escherichia coli* O157:H7) are able to survive and even grow in contaminated irrigation water [3,4], the reported numbers of food- and waterborne outbreaks are increasing. According to the European Food Safety Authority (EFSA) growing numbers of outbreaks, cases, hospitalizations, and deaths related to food of non-animal origin were observed [5]. Leafy vegetables irrigated with contaminated water are considered to be a common cause of human gastroenteritis, due to the presence of microbial pathogens. Turner et al. investigated a 21-year period (1996–2016) in the US in which 46 outbreaks caused approximately 2240 cases of illnesses with romaine lettuce and spinach as the affected vehicles [6]. Furthermore, EFSA reported 31 outbreaks related to vegetables and juices, resulting in 626 cases in 2018 and 48 waterborne outbreaks related to the consumption of tap water, including well water resulting in 1969 cases in 2019 [5,7].

In addition to being a reservoir for foodborne pathogens, soils, manure, and wastewater have been recognized as hot spots of antibiotic resistance gene (ARG) dissemination with antibiotic residues and ARGs being observed in each matrix [8,9,10,11]. For example, aquatic environments have been reported to be reservoirs of ARGs, such as colistin resistance-encoding element (*mcr-1*) in sewage and fresh water, resistance genes of tetracycline (TC) and sulfamethazine (SMZ) in irrigation water, and sulfonamide resistance genes (*sul1*, *sul2*) in raw sewage, treated effluent [11,12,13]. Therefore, the spread of antimicrobial resistance (AMR) and antimicrobial resistance genes (AGR) in the food production chain is also a global problem. For these reasons, the microbiological quality of irrigation water used in the food production chain, particularly leafy vegetables should be monitored to stop the increase of such cases.

Traditionally, bacteria have been identified by microbiological methods, e.g., assessing morphological and biochemical attributes of the isolates or more recently by molecular biology techniques such as PCR coupled with Sanger sequencing, targeting the 16S rRNA gene and pairing the gene sequences of isolates with classified references in generally known databases. GenBank (NCBI), one of the biggest and well-known databases, currently comprises more than 21,000,000 entries of bacterial 16S rRNA gene sequences with high-quality coverage derived from clinical and environmental settings. However, as these methods are generally based on the need of trained laboratory personnel and can take 3–5 days, they are not suitable for rapid bacterial identification and monitoring [14,15]. As an alternative, MALDI-TOF MS (matrix-assisted laser desorption ionization–time of flight mass spectrometry) has become a popular technique for microbiological identification in clinical settings due to its fast, less expensive, and labor-saving characteristics, compared to molecular identification techniques and biochemical-based tests. Identification based on MALDI-TOF MS measurements can either be performed by comparing the PMF (Protein Mass Fingerprint) of the measured microorganism to PMF databases, or by matching the masses of the identified biomarkers of unknown organisms utilizing proteomic databases [16]. The latest MALDI Biotyper (Bruker) library contains PMFs of 4274 unique bacterial species from 704 genera (2022). The majority of these PMFs are from clinical isolates and reference strains, which could be problematic for the identification of environmental isolates. In recent years, several authors tested the applicability of MALDI-TOF MS in environmental microbiology, but data on its efficacy in identifying waterborne microorganisms isolated directly from the environment is limited. Uchida-Fuji et al. showed the potential of MALDI-TOF MS (Bruker Biotyper) in environmental microbiology as the authors were able to identify 86.2% of 3724 isolates at species level [17]. In addition, MALDI-TOF MS (Bruker Biotyper) and 16S rRNA gene sequencing identification techniques have been compared in various environments such as environmental mining samples, high-altitude soil samples, soil samples, and fresh vegetables [18,19,20,21,22].

A common issue in environmental monitoring is that natural environments consist of a wide variety of microbial species, but 99% of bacteria are not culturable [22]. Thus, amplicon sequence analysis of marker genes such as the bacterial 16S rRNA gene is used to characterize the relative abundance of different bacterial genera and the entire bacterial community in environmental samples. This novel technology has been used to investigate the bacterial community of various aqueous environments [23,24,25,26,27,28].

In this study, we aimed to investigate the potential of MALDI-TOF MS to identify waterborne bacteria and to analyze the bacterial community of irrigation water in Eastern Hungary by using 16S rRNA gene amplicon sequencing. Our goal was to assess whether irrigation water in Eastern Hungary is a microbial risk in the food production chain and whether MALDI-TOF MS is a suitable tool for monitoring the microbiological quality of irrigation water. Analyzing the microbial community of irrigation water used for food production can mitigate the growing numbers of foodborne infections. 

## 2. Materials and Methods

Water samples were collected from wells used for irrigation from different towns located in Eastern Hungary in the county of Jász-Nagykun-Szolnok, such as Karcag, Kengyel, Rákóczifalva, Szolnok (two samples were taken from different sampling spots with the first one being artesian water). Water samples are marked as Sample 1 (Kengyel), Sample 2 (Karcag), Sample 3 (Rákóczifalva), Sample 4 (Szolnok1), Sample 5 (Szolnok 2). The sampling sites were chosen because the water is used for food production. Water samples were collected by holding sterile bottles directly under the water faucet to avoid microbial contamination and were transported to the lab under cooled conditions. Experiments were performed at University of Natural Resources and Life Sciences, Vienna (BOKU), Department of Food Science and Technology, Institute of Food Science. The MALDI-TOF MS instrument (Bruker MALDI Biotyper) was provided by the EQ-BOKU-VIBT GmbH.

### 2.1. Bacterial Isolation

Bacterial isolation was performed after preparing a ten-fold serial dilution in buffered peptone water (BPW) (Thermo Fisher Scientific Inc., Oxoid Ltd., Basingstoke, UK) up to dilution 10-3. The dilutions were plated in duplicates on Trypticase Soy Agar (TSA) (Merck Millipore, Burlington, MA, USA), Violet Red Bile Dextrose agar (VRBD) (Merck Millipore, Burlington, MA, USA), Reasoner’s 2A agar (R2A agar) (Merck Millipore, Burlington, MA, USA), and Yeast Extract Agar (Merck Millipore, Burlington, MA, USA) plates by spread plate method. VRBD plates were incubated at 37 °C for 24–48 h while plates with the other culture media were incubated at 30 °C for 24–48 h.

### 2.2. MALDI-TOF MS Identification

To identify the isolates, extended direct transfer procedure was used; therefore, each colony of isolates was placed onto the Bruker’s ground steel target plate, overlaid with 1 μL of 70% formic acid, and after the samples were air dried, 1 μL of α-cyano-4 hydroxycinnamic acid matrix solution (HCCA) was added. Each bacterial colony was measured two times. The identification process was completed using MALDI Biotyper 3.0. (Bruker Daltonics GmbH & Co., Billerica, MA, USA). MALDI-TOF MS spectra of the samples were collected using a Microflex LT/SH (Bruker Daltonics GmbH & Co., Bremen, Germany) mass spectrometer equipped with a nitrogen laser (lambda = 337 nm) at a laser frequency of 60 Hz operating in linear positive ion detection mode under MALDI Biotyper 3.0 Realtime classification (RTC) (Bruker Daltonics GmbH & Co., Bremen, Germany) and FlexControl 3.4 (Bruker Daltonics GmbH & Co., Bremen, Germany). Mass spectra were acquired in the range of 2000–21,000 Da for each sample analyzed for species-level microbial identification. MALDI-TOF MS spectra were generated from 240 single spectra that were created in 40-laser-shot steps from random positions of each isolate. The system was calibrated using *E. coli* ribosomal protein standard (Bruker IVD Bacterial Test Standard, Bruker Daltonics GmbH & Co., Bremen, Germany). FlexControl and FlexAnalysis (Bruker Daltonics GmbH & Co., Bremen, Germany) were used for data acquisition and data processing, respectively. FlexAnalysis was used to preprocess mass spectra which involves baseline subtraction, smoothing, and peak picking. 

MALDI-TOF MS identification results were accepted at genus or species level according to Bruker’s instructions. High-confidence identification indicates a score in the range of 2.00–3.00 which means reliable identification at species level. Low-confidence identification is accepted at genus level, with the score of 1.7–1.99. Scores below 1.7 are considered as not reliable identifications at any level. As for 16S rRNA gene sequencing, in accordance with previous findings, 98.65% sequence similarity threshold was accepted to bacterial species demarcation [20,29,30], and genus-level identification was obtained at 95% sequence similarity [31,32].

### 2.3. DNA Extraction and Sanger Sequencing of Waterborne Isolates

DNA extraction of the previously cultured isolates was performed by Chelex Method. Chelex solution contained 2.5 g Chelex (Bio-Rad Laboratories, Hercules, CA, USA), 2.5 mL 0.01 M Tris HCL and 95 mL distilled water (Thermo Fisher Scientific, Waltham, MA, USA). A colony of each isolate was put into 500 μL Chelex solution with a sterile inoculation loop. After mixing by vortexing, the samples were placed into Eppendorf ThermoMixer C (Eppendorf, Hamburg, Germany) and incubated for 10 min at 95 °C. Then samples were centrifuged at 15,000× *g* for 30 s and the supernatant was transferred into a fresh 2 mL Eppendorf tube. After extracting the DNA of isolates, 16S rRNA gene specific PCR was performed. The applied 16S rRNA gene primers were 27F, 5′-AGAGTTTGATCCTGGCTCAG-3′ and 1492R, 5′-GGTTACCTTGTTACGACTT-3′. The PCR thermal profile was set to 95 °C for 5 min, followed by 30 cycles of 94 °C for 30 s, 55 °C for 30 s, and 72 °C for 1 min, and concluded with a final elongation step at 72 °C for 10 min. PCR products were evaluated by 1% agarose gel electrophoresis. Samples were purified with the peqGOLD Cycle-Pure Kit (VWR International, Radnor, PA, USA) following the manufacturer’s instructions. Then, 3 μL of 27F gene primer was added to 12 μL of purified DNA, then DNA Sanger sequencing was performed by Microsynth AG (Balgach, Switzerland). Sequences of the isolates were blasted against the NCBI RefSeq RNA sequence database to identify them.

### 2.4. DNA Extraction and Next-Generation Sequencing of Irrigation Water Samples

For the isolation of microbial genomic DNA from irrigation water samples, DNeasy PowerSoil Pro Kit (QIAGEN, Hilden, Germany) was used. The procedure was performed following the manufacturer’s instructions. First, 250 μL of each sample was added to a dry bead tube with garnet beads from the DNA isolation kit. Next, 800 μL of bead solution was added to the samples to disintegrate the cell walls. The samples were vortexed for 10 min. After centrifugation at 15,000× *g* for 1 min, the supernatant was transferred into a 2 mL collection tube. Then, 200 μL of the respective solution of the isolation kit were added, and it was vortexed for 5 s to precipitate non-DNA organic and inorganic material. The tubes were centrifuged at 15,000× *g* for 1 min, and the supernatant was transferred into a clean 2 mL microcentrifuge tube. It was followed by adding 600 μL of a high-concentrate salt solution then 5 s of vortexing was performed. After that, 650 μL of the lysate was loaded to an MB spin column and centrifuged for 1 min at 15,000× *g*. Flow-through was discarded, and the column was centrifuged again. The MB spin column was transferred to a clean collection tube, and 500 μL of a wash solution was added, followed by a centrifugation step. This step was repeated with 500 μL of an ethanol-based wash solution to further clean the DNA and allowing it to stay bound to the silica membrane. Flow-throughs were discarded after each centrifugation. Centrifugation for 2 min at 15,000× *g* ensured the absence of remaining washing solutions. The column was placed into a new 1.5 mL elution tube. Then, 50 μL of elution buffer was placed on the column, and DNA was eluted via centrifugation for 1 min. Amplicon library generation, quality control, and sequencing were performed at the Vienna Biocenter Core Facilities NGS Unit (www.vbcf.ac.at, accessed on 20 January 2023). The V3–V5 hypervariable region of the 16S rRNA gene was amplified and sequenced using a MiSeq Illumina platform with a 300 bp paired-end read protocol (Illumina, Inc., San Diego, CA, USA). The PCR reactions were performed as described in Klindworth et al. using the forward primer 341f 5′-TCGTCGGCAGCGTCAGATGTGTATAAGAGACAG and the reverse primer 785r 5′-GTCTCGTGGGCTCGGAGATGTGTATAAGAGACAG [33].

### 2.5. Bioinformatics and Data Processing of Next-Generation Sequencing Data

Primers were removed from the raw sequences using cutadapt v2.1 [34]. Raw sequences were further processed with the dada2 v1.14.1 pipeline in R v3.6.3 [35,36]. Briefly, low-quality sequences were filtered using ‘filterAndTrim’ with a maximum number of expected errors of 2 and trimming set at a length where the quality score dropped below 30. After learning the error rates with the ‘learnErrors’ command, samples were dereplicated using ‘derepFastq’ and the dada 2 sample inference algorithm was run with default parameters. Then, forward and reverse reads were merged with the ‘mergePairs’ command, choosing a minOverlap = 10 and a maxMismatch = 1. ASV tables were constructed with the ‘makeSequenceTable’ command. Chimeric sequences were removed using the ‘removeBimeraDenovo’ command with the consensus method. Taxonomic assignment was performed via the SILVA rRNA database SSU 138 using the ‘assignTaxonomy’ command [37]. 

### 2.6. Statistical Methods

A paired t-test was used to compare the efficacy of identification of the MALDI-TOF MS and 16S rRNA gene sequencing of isolates (IBM SPSS Statistics 27, Armonk, NY, USA). MicrobiomeAnalyst was used to analyze data derived from 16S rRNA amplicon sequencing [38,39]. A total of 33 low-abundance ASVs were removed based on low prevalence (set at 20%) and low count (<4). After data filtering step, 730 ASVs were used for further analysis and included in the results. Data were normalized by total sum scaling (TSS), i.e., the number of reads from the same ASV were divided by the total number of reads in each sample. Hierarchical Clustering and Heatmap visualization were based on Euclidean distance with the application of Ward clustering algorithm.

## 3. Results

### 3.1. Results of MALDI-TOF MS Identification and Sanger Sequencing of Isolates

The applied methods generated similar identification results (Table 1). Both 16S rRNA gene sequencing and MALDI-TOF MS identified more than 60% of the 42 waterborne isolates similarly at species level. However, the application of MALDI-TOF MS made it possible to identify more isolates at species level. At genus-level identification, a minor difference was observed as MALDI-TOF MS could identify more isolates properly. However, a paired t-test showed that the identification results of the two methods did not differ significantly t(41) = 2.02; *p* = 0.57).

Most isolates identified by MALDI-TOF MS were categorized as Gram-negative bacteria. The most frequently cultivated isolates belonged to genus *Acinetobacter*, *Enterobacter*, *Pseudomonas,* and *Brevundimonas*. MALDI-TOF MS failed to identify two isolates at any level, but those were categorized as a *Pseudomonas stutzeri* and a *Sphingobacterium kitahiroshimense* isolates with high-confidence by Sanger sequencing. Four isolates, belonging to genera *Acinetobacter*, *Pseudarthrobacter,* and *Stenotrophomonas*, were not identified properly even at genus level by Sanger sequencing. The first isolate was identified as *Acinetobacter ursingii* with high confidence by MALDI-TOF MS; however, with Sanger sequencing, only 90.41% similarity was obtained. The next isolate was also identified as an *Acinetobacter ursingii* isolate with low-confidence by MALDI-TOF MS while with Sanger sequencing 91.67% similarity was achieved. The third isolate was identified as a member of *Pseudarthrobacter* at genus level by MALDI-TOF MS, and it was related to a *Pseudarthrobacter siccitolerans* isolate with 89.91% similarity by Sanger sequencing. The fourth isolate was identified as *Stenotrophomonas maltophilia* by MALDI-TOF MS with high confidence, but only 94.14% similarity was achieved by Sanger sequencing.

The most dominant genus was *Acinetobacter* as 20 isolates belonged to that genus. Four of twenty *Acinetobacter* isolates were only identified at genus level by MALDI-TOF MS, while sixteen were identified at species level. Similarly, 16 *Acinetobacter* isolates were identified at species level by 16S rRNA gene sequencing, while 2 isolates were only identified at genus level and 2 more not at all. One isolate was identified with low confidence as *Acinetobacter schindleri* by MALDI-TOF MS, while it was identified at species level similarly by Sanger sequencing.

The application of both methods resulted in similar outcomes in terms of identifying *Enterobacter* isolates. MALDI-TOF MS identified two *Enterobacter* isolates, one as *Enterobacter hormaechei* while the other being *Enterobacter cloacae*. Three isolates could not be identified at species level, because they had identical species identification scores for multiple species in both methods.

Isolates belonging to the genus *Pseudomonas* were also frequent, as five isolates were categorized into it. One isolate could not be identified by MALDI-TOF MS, but it was identified by Sanger sequencing as its sequence had 99.64% similarity score with sequences of *Pseudomonas stutzeri*. In three cases, Sanger sequencing could not differentiate *Pseudomonas* species correctly; however, two of those isolates were identified as *Pseudomonas veronii* with high confidence by MALDI-TOF MS. One isolate was identified correctly at species level as *P. stutzeri* by both methods.

Eleven of the 42 isolates were identified differently by MALDI-TOF MS and 16S rRNA gene sequencing (Table 2). Only two isolates were identified differently of the genus *Acinetobacter*, the most commonly found genus. In both cases, MALDI-TOF MS identification resulted in *Acinetobacter junii*, whereas those isolates were identified as *Acinetobacter schindleri* by Sanger sequencing. An isolate, identified as *Rhodococcus erythropolis* with low confidence by MALDI, was identified as *Rhodococcus qinsengii* by Sanger sequencing. Interestingly, neither of the techniques were able to identify this isolate at species level with high confidence. 

Discrepancies were also found among *Enterobacter* and *Pseudomonas* isolates. Isolates marked as #6 and #9 were identified as *E. hormaechei* and *E. cloacae* with high confidence by MALDI; however, Sanger sequencing could not differentiate the former as sequences of both *E. cloacae* and *E. hormaechei* showed 99.9% similarity. Although isolate #9 was identified as *E. cloacae* with high confidence, it was identified as *E. hormaechei* by Sanger sequencing with 99.48% similarity. Isolates marked as #10 and #11 were identified as *Pseudomonas veronii* with high confidence by MALDI-TOF MS, while the former was identified as *P. veronii*/*P. extremaustralis* showing 100% similarity scores for both species by Sanger sequencing. The best-matched hit for the latter was an uncharacterized *Pseudomonas* species.

### 3.2. Next-Generation Sequencing of Irrigation Water

**Figure 1:**
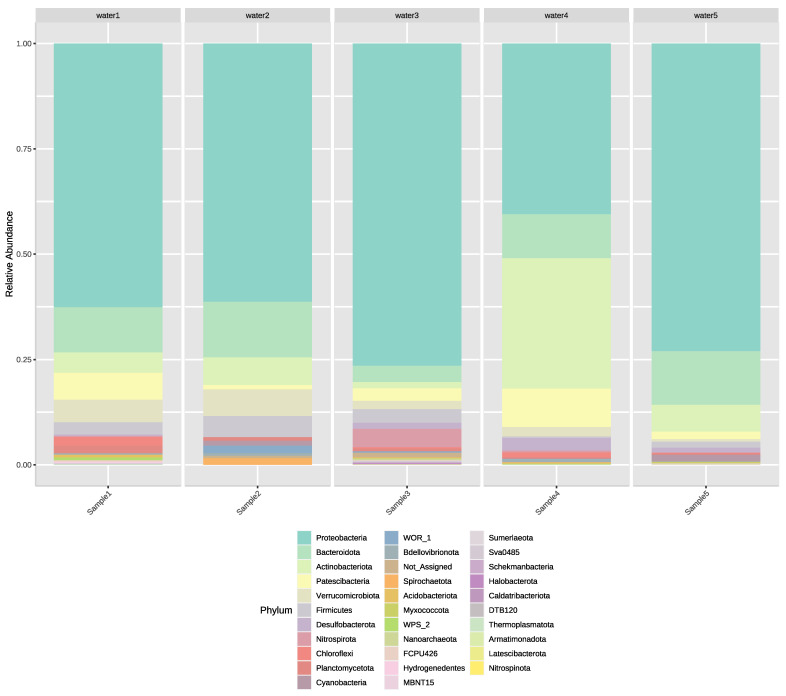
Relative abundance of ASVs regarding each irrigation water sample. ASVs are shown on phylum level.

The relative abundance of ASVs shows a wide range of variety at phylum level displaying 32 different phyla (Figure 1). Altogether, 730 ASVs were found in the five samples which comprised 82,613 total read counts. On average, 229 high quality 16S rRNA gene sequences per sample remained after stringent quality filtering.

Sample 1 contained the most read counts with 20,785, and it was followed by Sample 3 with 19,637 read counts. Sample 4 comprised 18,368 while Sample 5 contained 13,086 read counts. The lowest number of read counts, only 10,737, was found in Sample 2. A rarefaction curve indicated that all samples were sequenced deep enough to infer the full diversity of microorganisms in the samples (Supplementary file S1). The species richness ranged from 106 ASVs in Sample 2 to 392 ASVs in Sample 4, whereas the Shannon index ranged from 4.02 to 4.99 (Supplementary file S2).

The most abundant phylum was Proteobacteria followed by Actinobacteria, Bacteroidota, Patescibacteria, and Verrucomicrobiota in the analyzed samples (Figure 1). However, differences were observed regarding the abundance of phyla in each sample. In Sample 1, Proteobacteria (63%) were most abundant, while the other most common phyla were Bacteriodota (11%), Patescibacteria (6%), Verrucomicrobiota (5%), and Actinobacteriota (5%). The abundance of phyla found in Sample 2 was similar to Sample 5 as in both samples Proteobacteria (61%; 73%) was followed by Bacteroidota (13%; 13%), Actinobacteria (7%; 6%). Sample 4 differed from other samples, as most of the ASVs belonged to Proteobacteria (40%) while ASVs from Actinobacteria (31%) were also common, followed by ASVs belonging to Bacteroidota (10%), Patescibacteria (9%), and Desulfobacteria (3%). The dominance of Proteobacteria could also be observed in both Sample 3 (76%) and Sample 5 (73%). However, composition of these samples was divergent as in Sample 3 the next most abundant phyla were Nitrospirota (4%), Bacteriodota (4%), Firmicutes (3%), and Patescibacteria (3%), whereas in Sample 5 the second most abundant phylum, Bacteriodota (13%), was followed by Actinobacteria (6%), Patescibacteria (2%), and Cyanobacteria (2%).

**Figure 2:**
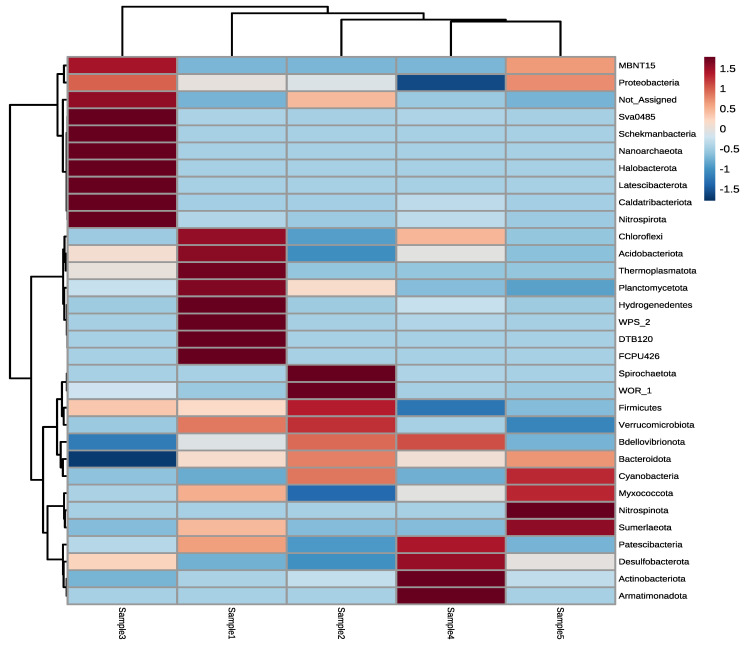
Taxonomic distribution of the phylogenetic groups at phylum level of irrigation water samples shown by combining Hierarchical Clustering and Heatmap visualization.

The taxonomic distribution of phylogenetic groups of irrigation waters shows specific fingerprints regarding bacterial phyla in each sample (Figure 2). The application of Hierarchical Clustering classified the samples into clusters based on their microbial communities. Sample 3 can be distinguished from other samples because phyla including MBNT15, Sva0485, Schekmanbacteria, Nanoarchaeota, Halobacterota, Latescibacterota, Caldatribacteriota, and Nitrospirota are mainly abundant in only that sample. In contrast, Sample 4 and Sample 5 are phylogenetically more related to each other. The cluster of Sample 4 and Sample5 can be extended by Sample 2, and the cluster of these three samples can be further extended by adding Sample 1. Therefore, two great clusters can be differentiated with one containing only Sample 3 while the other contains the rest of the samples (Sample 1, Sample 2, Sample 4, and Sample 5).

**Figure 3:**
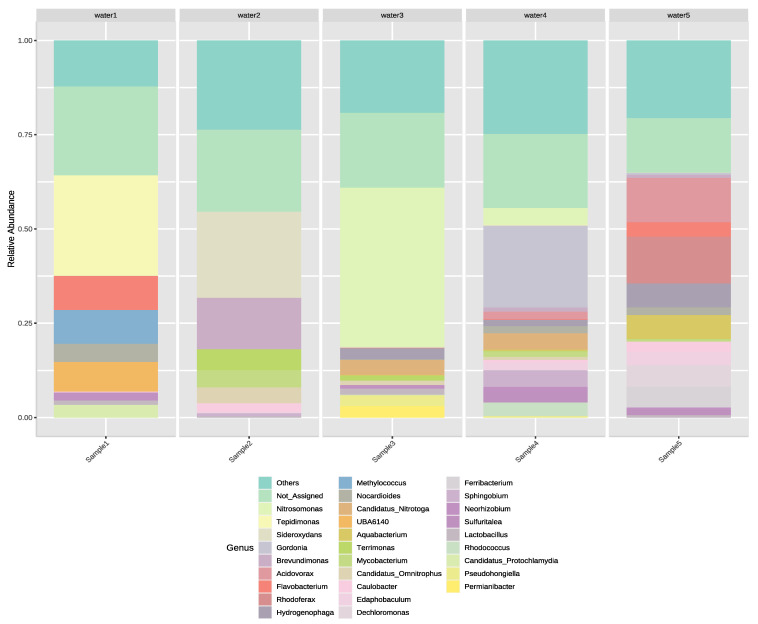
Relative abundance of the 30 most abundantly occurring bacterial genera in the irrigation water samples. The rest of the taxa merged into the group of others.

The microbial community of water samples was diverse as the five most abundant genera were different in each sample (Figure 3). In Sample 1, the most abundant genus was *Tepidimonas* followed by *Flavobacterium*, *Methylococcus*, *Methylophilaceae* UBA6140, and *Nocardioides*. In Sample 2, the most abundant genus was *Sideroxydans,* which was followed by genus *Brevundimonas*, *Terrimonas*, *Mycobacterium*, and *Candidatus_Omnitrophus*. In Sample 3, the most abundant genus was the ammonia-oxidizing *Nitrosomonas* which was followed by *Candidatus Nitrotoga*, an uncultured nitrite-oxidizing and naturally occurring bacterial genus in aqueous ecosystems [40], and *Permianibacter*. Genus *Hydrogenophaga*, a hydrogen oxidizing genus, and *Pseudohongiella*, of which species have been isolated from seawater [41], were also common. The abundance of genera detected in Sample 4 was similar to Sample 3 as nitrifying-bacterial genera such as *Nitrosomonas* and *Candidatus Nitrotoga* were the second and fourth most abundant genera. However, the most abundant genus was *Gordonia* and the third most abundant genus was *Sphingobium* while the fifth was genus *Rhodococcus*. In Sample 5, the dominance of Comamonadaceae family could be observed as four of the five most abundant genera belonged to that family. The most abundant genus was *Rhodoferax* followed by *Acidovorax*, *Hydrogenophaga*, *Aquabacterium,* and *Dechloromonas*.

Although genus *Nitrosomonas* was the most dominant in terms of relative abundance (11.04%), zero isolates were cultivated from it. In contrast, *Acinetobacter*, the most dominant genus regarding cultivated isolates, was only the 31st in terms of relative abundance (0.64%) in the entire bacterial community (Table 3). Similarly, despite the fact that five isolates had been isolated from genera *Pseudomonas* and *Enterobacter*, their relative abundance was only 0.24% and 0.04%, respectively. Species of the genus *Brevundimonas*, the genus with the highest relative abundance (2.18%) among cultivated genera, were cultivated three times. Furthermore, two isolates of the genus *Rhodococcus* were cultivated, which also had the second highest relative abundance (0.81%) value among cultivated genera. Although only one isolate was cultivated and identified as a member of the genus *Chryseobacterium,* its relative abundance (0.35%) was the fourth highest among cultivated genera.

## 4. Discussion

The applicability of MALDI-TOF MS and its databases (Bruker Biotyper, VITEK MS) have been studied and validated for clinical microbiology laboratories in recent years [42,43,44,45,46]. However, its application regarding isolates derived from the food production chain and its environment is challenging due to the microbial diversity in soil and water matrices which contain thousands of different bacterial species [47,48]. Strejcek et al. applied both MALDI-TOF MS (Bruker Biotyper) and 16S rRNA gene sequencing to identify microorganisms found in soils and sediments and obtained concordant genus-level identification (92%) while at species level only 35% of the isolates identified coincided with those identified by 16S rRNA gene sequencing analysis [20]. Kopcakova et al. used MALDI-TOF MS (Bruker Biotyper) to identify the microflora from waste disposal sites with an overall identification rate lower than 20% at species level [49]. However, Suzuki et al. used MALDI-TOF MS (Bruker Biotyper) to identify coliform bacteria from sewage, river water, and groundwater, obtaining identical results at genus level in 96%, 74%, and 62% of the isolates, respectively, compared to 16S rRNA gene sequence analysis [50].

In congruence with the study of Suzuki et al., our results indicate that MALDI-TOF MS can be used to identify waterborne bacterial isolates, as more isolates were identified at species level than with 16S rRNA gene sequencing [50]. MALDI-TOF MS generated 95.24% correct genus level identification of the cultivated 42 isolates which were higher than the results of 16S rRNA gene sequencing (90.48%). Moreover, 73.81% of the isolates were identified identically with both methods. Four of the isolates (9.52%) were not identified at any level by 16S rRNA gene sequencing while the number of unidentified isolates were only two (4.76%) with MALDI-TOF MS.

Similarly, Böhme et al. compared the efficacy of MALDI-TOF MS (Voyager STR-DE, Applied Biosystems) and 16S rRNA gene sequencing and pointed out that MALDI-TOF MS identified 76% of 50 seafood-borne bacterial strains isolated from commercial seafood products at species level while 16S rRNA gene sequencing only identified the species of 50% of the strains [51]. Our outcomes are also consistent with the study of El-Nemr et al. in which MALDI-TOF MS identified more bacteria isolated from a market area (e.g., vegetables, soil, air, and hand palms of fresh produce handlers) at species level (41%) than 16S rRNA gene sequencing (28%) did [21]. In another study, Pandey et al. identified psychrotolerant bacteria isolated from high-altitude soil with only 4.92% of the isolates identified similarly by MALDI-TOF MS and 16S rRNA gene sequencing [19], whereas in our case 73.81% of the isolates were identified identical. Our finding is close to the study of Avanzi et al. in which 82% of the copper resistant microorganisms, isolated from environmental mining samples (soil and water), were identified similarly with both methods [18]. Moreover, in the aforementioned study of Pandey et al. 19.67% of the isolates were not identified at any level by MALDI-TOF MS which value is higher than the result (4.76%) obtained in our study [19]. One fact which could have contributed to the lower scores of the Pandey et al. study is that at the time of its conduction some of the unidentified isolates (*Bacillus wiedmannii*, *Bacillus velezensis*, *Bacillus paramycoides*) were not included in the database.

In our study, most of the cultivable isolates belonged to the genus *Acinetobacter*. In terms of identifying species of this genus, both methods could achieve almost identical results as 16 isolates were identified at species level. MALDI-TOF MS (Bruker Biotyper) outperformed 16S rRNA gene sequencing as the former identified four isolates at genus level, while the latter, besides identifying two isolates at genus level, was not able to identify two isolates at any level. Species of this genus have been found in agricultural and hydrocarbon-polluted soils, water, sediment, industrial wastewater, and sewage [52]. Most of the *Acinetobacter* isolates were identified as *A. junii* which has been previously reported to be found in aquatic environments such as surface water, sewage, marine sediments, and wastewater [52,53,54,55,56]. Other isolates were identified as *A. schindleri*. This species has been isolated from other sources before, for example livestock animals and pets, head lice from primary school pupils, or from soil samples [57,58,59]. Another species, already isolated from raw meat and human fecal samples [60,61], *Acinetobacter ursingii* was also found in our irrigation water samples. 

Our results and the findings of several other authors reassure the potential of MALDI-TOF MS (Bruker Biotyper) to act as an alternative to 16S rRNA gene sequencing or in the future even replace it in environmental microbiology screening [17,18,19,20,21,50,51]. Still, to identify and thoroughly characterize the bigger, uncultivable part of the microbial community of irrigation water, culture-independent methods such as amplicon sequence analysis of 16S rRNA genes are necessary. Our results from the next-generation sequencing approach showed a dominance of Proteobacteria followed by Actinobacteria, Bacteroidota, Patescibacteria, Verrucomicrobiota, and Firmicutes. In the samples, Proteobacteria (62%) was by far the most dominant while Actinobacteria (10%), Bacteriodota (10%), Patescibacteria (5%), Verrucomicrobiota (3%), and Firmicutes (2%) occurred less frequently. It is also notable that Actinobacteria were more abundant in Sample 4 (32%) compared to the other samples (<10%). This sample was the only artesian water sample included in the study.

Jin et al. analyzed the microbial community characteristics of 16 surface water samples in the Beijing area using 16S rRNA gene amplicon sequencing and found that Proteobacteria and Bacteroidetes were the most commonly identified phyla in all the samples, accounting for 21.9–78.5% and 19.1–74.7% of the sequences, respectively [26]. Lehosmaa et al. studied the bacterial communities of groundwater–surface water ecotone of boreal springs and observed that the bacterial communities were dominated by Proteobacteria (50%) based on relative abundance, followed by Bacteroidetes (18%), Patescibacteria, and Acidobacteria (4% each) [61]. In the study of Jin et al. the most predominant genera among Proteobacterial sequences were *Hydrogenophaga* and *Rhodoferax* both of which were found to be dominant in our samples as well [26]. The former was dominant in both Sample 3 and Sample 5 whereas the latter in Sample 5. Moreover, genus *Hydrogenophaga* had both high relative abundance and prevalence in our samples. Genus *Flavobacterium*, the predominant Bacteroidetes genus in the aforementioned study of Jin et al. [26], had also high relative abundance in our study. Iliev et al. applied 16S rRNA gene amplicon sequencing to analyze microbial freshwater communities in two Bulgarian reservoirs and found that Proteobacteria, Actinobacteria, and Bacteroidetes contained more than 95% of the relative abundance, regardless of the reservoir’s large hydrogeological differences [24]. These findings are in line with our results, suggesting that Proteobacteria and Bacteroidetes are among the dominant phyla in different water bodies across the globe [24,26,62]. 

Nitrite-oxidizer bacteria were not isolated but were common in our samples as *Nitrosomonas* was the most abundant genus in Sample 3 and the second most abundant in Sample 4. Moreover, *Nitrotoga*, a main nitrite-oxidizer in activated sludge systems with nutrient removal [23], was the second most abundant genus in Sample 3 and the fourth most abundant in Sample 4. The isolates were dominated by the genus *Acinetobacter*, although it only had the third highest relative abundance value among cultivated genera with the first one being genus *Brevundimonas*. Furthermore, while having cultivated several *Enterobacter* and *Pseudomonas* isolates in our study, two ubiquitous and potentially pathogenic genera, ASVs belonging to any of those genera had a low relative abundance in the samples. The majority of the cultivated genera (8 of 12) had a relative abundance of at least 0.01% in the amplicon dataset. Moreover, only 8 of 188 (4.25%) genera, which had a relative abundance above 0.01% were cultivated. Together, this highlights the fact that most of the environmental bacteria are uncultivable. 

In the present study, it was successfully shown that MALDI-TOF MS (Bruker Biotyper) can act as an alternative to 16S rRNA gene sequencing of isolates to identify waterborne bacteria due its rapid and accurate nature. As MALDI-TOF MS identification relies on matching the PMF of the measured isolate to the database, lack of entries will lead to misidentifications or not reliable identifications. However, as more species’ mass spectra are being generated and added into the commercially available mass spectral databases, the environmental applicability of MALDI-TOF MS will be even further improved. However, MALDI-TOF MS identification of isolates should be coupled with next-generation sequencing to also determine the uncultivable part of the microbial community and low abundant pathogens that might escape cultivation. Together, the two methods are suitable to analyze the microbial community composition of irrigation water used in food production and to determine whether the water constitutes a potential risk to the food production chain. 

In conclusion, monitoring and characterizing the bacterial diversity of irrigation water with MALDI-TOF MS and NGS can help in preventing and reducing foodborne infections.

## Figures and Tables

**Table 1:** Identification results of 16S rRNA gene Sanger sequencing and MALDI-TOF MS regarding each waterborne isolate shown at genus level.

		16S rRNA Gene Sequencing Identification			MALDI-TOF MS Identification		
Bacterial Genus	Number of Isolates	Species Level > 98.5%	Genus Level > 95%	No Identification < 95%	Species Level > 2	Genus Level 2 > 1.7	No Identification <1.7
*Acinetobacter*	20	16	2	2	16	4	
*Aeromonas*	1	1			1		
*Brevundimonas*	3	3			3		
*Chryseobacterium*	1	1			1		
*Enterobacter*	5	1	4		2	3	
*Microbacterium*	1	1			1		
*Pantoea*	1		1		1		
*Pseudarthrobacter*	1			1		1	
*Pseudomonas*	5	2	3		3	1	1
*Rhodococcus*	2	1	1			2	
*Sphingobacterium*	1	1					1
*Stenotrophomonas*	1			1	1		
*Total isolates*	42	27 (64.3%)	38 (90.5%)	4 (9.5%)	28 (66.7%)	40 (95.2%)	2 (4.8%)

**Table 2:** Differently identified isolates by MALDI-TOF MS and 16S rRNA gene sequencing.

No.	Isolate	MALDI-TOF MS Identification (Log Score, Consistency Category)	16S rRNA Identification (% Similarity Score)
#1	Sample5/9	*Acinetobacter junii* (2.34; A)	*Acinetobacter schindleri* (99.24%)
#2	Sample5/12	*Acinetobacter junii* (2.1; A)	*Acinetobacter schindleri* (98.78%)
#3	Sample3/1	*Rhodococcus* spp. (1.71; B)	*Rhodococcus qinsenghii* (96.2%)
#4	Sample3/3	No ID (1.51; C)	*Sphingobacterium kitahiroshimense* (99.72%)
#5	Sample3/4	*Chryseobacterium indologenes* (2.01; A)	*Chryseobacterium lactis* (98.8%)
#6	Sample2/4	*Enterobacter hormaechei* (2.25; A)	*Enterobacter cloacae/E. hormaechei* (99.9%)
#7	Sample2/5	*Pseudarthrobacter scleromae/oxydans* (2.24; B)	*Pseudarthrobacter siccitolerans* (89.91%)
#8	Sample2/6	*Rhodococcus* spp. (1.99; B)	*Rhodococcus cerastii* (99.46%)
#9	Sample2/7	*Enterobacter cloacae* (2.27; A)	*E. hormacheai* (99.48%)
#10	Sample2/8	*Pseudomonas veronii* (2.26; A)	*P. veronii/ P. extremaustralis* (100%)
#11	Sample2/9	*Pseudomonas veronii* (2.2; A)	*Pseudomonas* spp. (99.34%)

**Table 3:** Identified bacterial isolates and their relative abundance in the next-generation sequencing dataset.

Bacterial Genus	Number of Isolates	Relative Abundance of the Genera
*Brevundimonas*	3	2.18%
*Rhodococcus*	2	0.81%
*Acinetobacter*	20	0.64%.
*Chryseobacterium*	1	0.35%
*Pseudomonas*	5	0.24%
*Enterobacter*	5	0.04%
*Stenotrophomonas*	1	0.03%
*Sphingobacterium*	1	0.02%
*Aeromonas*	1	<0.01%
*Microbacterium*	1	<0.01%
*Pantoea*	1	<0.01%
*Pseudarthrobacter*	1	<0.01%

## Data Availability

The datasets generated during and/or analyzed during the current study are available from the corresponding author on request. Raw sequence data is available in the European Nucleotide Archive under accession number PRJEB56665.

## Supplementary Materials

The following supporting information can be downloaded at: https://www.mdpi.com/article/10.3390/microorganisms11020287/s1, File S1: Rarefaction curve regarding the water samples. File S2: Species richness and Shannon index values of the water samples.
