# Supplementary figures and images for: Comparing the Efficacy of MALDI-TOF MS and Sequencing-Based Identification Techniques (Sanger and NGS) to Monitor the Microbial Community of Irrigation Water
Source: Microorganisms. 2023 Jan 21;11(2):287. doi: 10.3390/microorganisms11020287 (PMC9960253; doi:10.3390/microorganisms11020287)

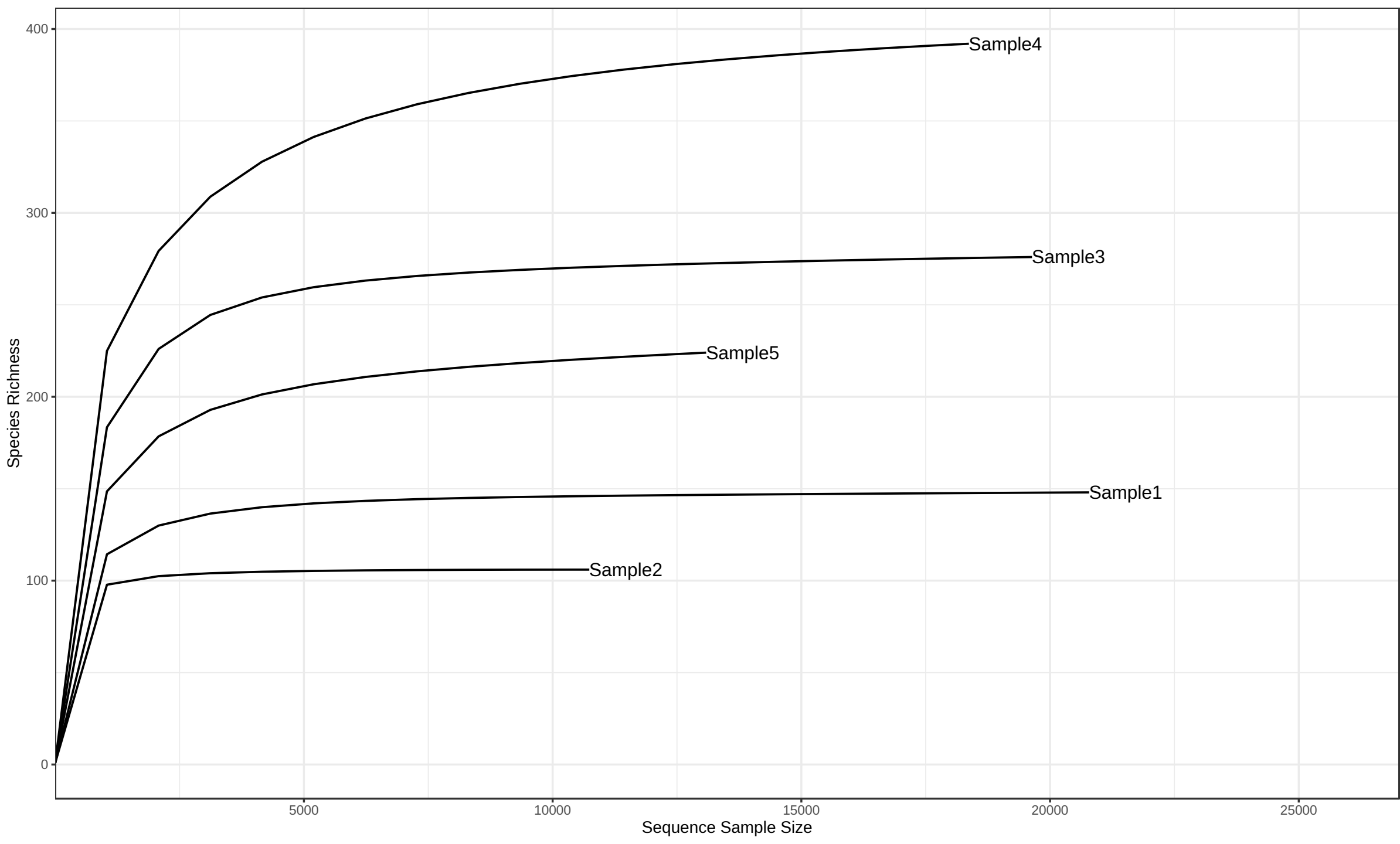

Supplement: Supplementary file 1 [file microorganisms-11-00287-s001.zip › Supplementary_file1.pdf]
